# Macular assessment of preoperative optical coherence tomography in ageing Chinese undergoing routine cataract surgery

**DOI:** 10.1038/s41598-018-22807-7

**Published:** 2018-03-23

**Authors:** Xiaoli Huang, Zhengwei Zhang, Jie Wang, Xiaomei Meng, Tiantian Chen, Zhifeng Wu

**Affiliations:** Department of Ophthalmology, Nanjing Medical University Affiliated Wuxi Second Hospital, Wuxi, Jiangsu Province P. R. China

## Abstract

This retrospective consecutive case series aimed to evaluate spectral-domain optical coherence tomography (SD-OCT) for occult macular disease recognition preoperatively in patients scheduled for routine cataract surgery. All patients scheduled for cataract surgery underwent macular SD-OCT. Scans were reviewed for retinal, retinal pigment epithelium and vitreomacular interface abnormalities. For the subgroup analysis, the following information was collected: age; sex; and diabetes, hypertension, myopia, glaucoma, post intra-ocular surgery, endophotocoagulation, retinal vasculopathy and uveitis statuses. One-thousand-one-hundred-seventy-six consecutive scans were acquired from 1,176 patients. Macular pathology was found in 294 eyes. The most common macular disorders were an epiretinal membrane (n = 130), myopia atrophy (n = 61) and a dome-shaped macular with pathologic myopia (n = 32). One-hundred-thirty eyes (11.05%) presented macular epiretinal membranes not detected by dilated fundus examination, accounting for 44.22% of the abnormalities in diseased eyes and was higher than in previous Chinese studies. Some had multiple macular disorders. The most common ocular history was myopia, including high myopia. The pooled prevalence rate of macular diseases detected by OCT was 0.24 (95% CI 0.14–0.34) using meta-analysis. SD-OCT should be performed for routine cataract surgery patients to evaluate visual outcomes, especially in myopic patients and those considering advanced-technology intraocular lenses.

## Introduction

China has a population of 1.3 billion accounting for 15% of the world’s population. The estimated prevalence of blindness in individuals aged ≥50 years was 2.3% in 2010^1^. The prevalence of low vision and blindness in Chinese adults remains a severe public health concern. Cataract was the primary cause of visual impairment in adults aged ≥45 years. For example, in the Taizhou Eye Study, cataract was the leading cause of low vision and blindness^2^.

Phacoemulsification with intraocular lens (IOL) implantation is the only effective treatment for patients with cataract and is the most commonly performed clinical procedure in the medical field^3^. Cataract surgery has made significant advancements in the past few decades, resulting in improved refractive outcomes and greater patient satisfaction. Suboptimal visual outcomes after uneventful cataract surgery can cause substantial patient dissatisfaction, especially in cases of missed macular pathology^4^. As approximately 2,000 patients undergo cataract surgery at our hospital each year, improvements in understanding the visual prognosis and management of these cases could confer a substantial benefit to both patients and surgeons. Identification of macular diseases preoperatively, especially in the presence of media opacities, remains challenging and important^4–6^. Spectral-domain optical coherence tomography (SD-OCT) is a noninvasive and sensitive test for evaluating the macular structure^7^. In order to recognize occult macular diseases preoperatively in patients scheduled for routine cataract surgery, we evaluated the macular structures of all patients before cataract surgery with SD-OCT.

## Results

**Figure 1:**
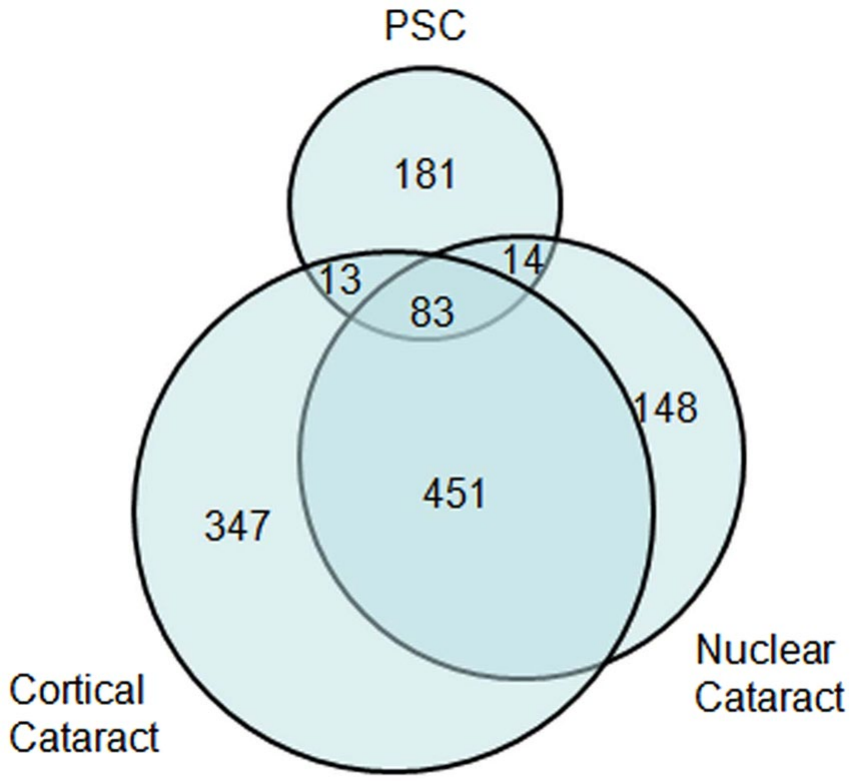
Venn diagram describing 992 eligible subjects with nuclear, cortical, and PSC cataracts.

**Figure 2:**
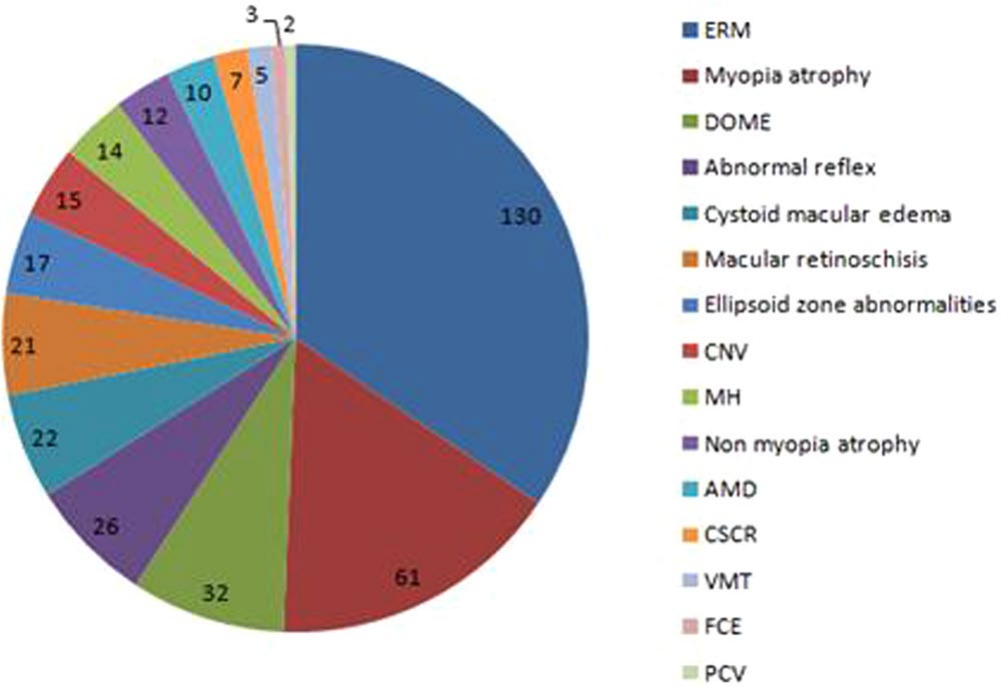
Frequencies of macular pathologies using SD-OCT.

**Figure 3:**
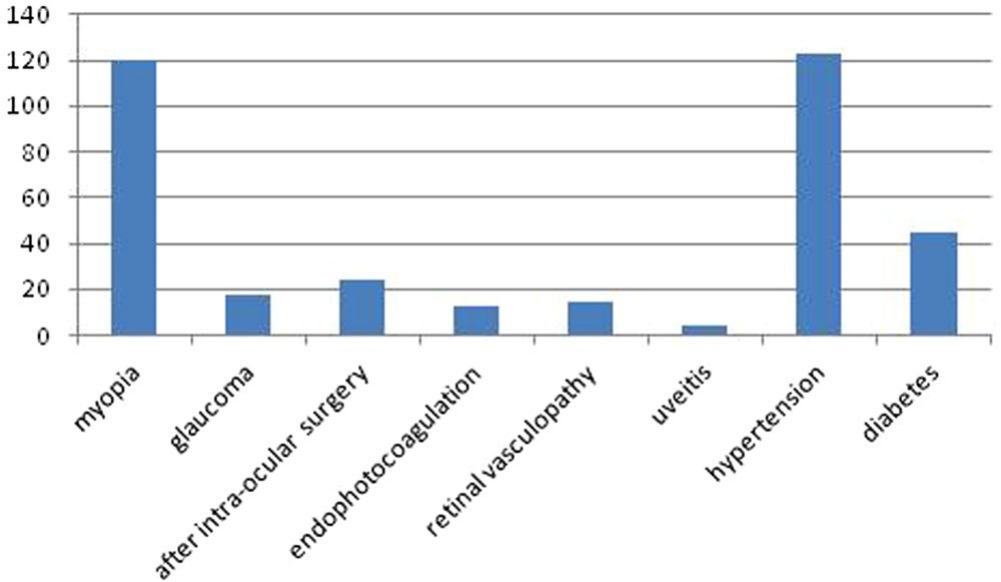
Systemic and ocular history distributions.

All 1,176 eyes in the 1,176 patients scheduled for routine cataract surgery during the study underwent a preoperative SD-OCT scan, of whom 685 were women. The mean age of the included patients was 69.39 ± 10.20 years (range, 40 to 95 years). Poor scan quality because of hypermature cataract precluded the analysis of 184 scans (15.65%). The type and the grade of cataract were assessed by the same experienced ophthalmologist according to the Lens Opacities Classification System III grading system and shown in Table 1. For the 992 eligible subjects with cataract, the relationship between nuclear, cortical, and PSC cataract was illustrated using a Venn diagram (Fig. 1). Macular pathology was observed in 294 eyes (25%) and normal scans were observed in 698 eyes (59.35%). The confirmed pathologies included epiretinal membrane (ERM, n = 130), myopic atrophy (n = 61), a dome-shaped macula (DSM) with pathologic myopia (n = 32), abnormalities in subfoveal and parafoveal reflexes (n = 26), cystoid macular oedema (n = 22), macular retinoschisis (n = 21), ellipsoid zone abnormalities (n = 17), choroidal neovascularization (CNV, n = 15), macular hole (MH, n = 14), non-myopic atrophy (n = 12), age-related macular degeneration (n = 10), central serous chorioretinopathy (n = 7), vitreomacular traction syndrome (n = 5), focal choroidal excavation (n = 3) and polypoidal choroidal vasculopathy (n = 2) (Fig. 2). Ellipsoid-zone abnormalities included interruptions or indentations in subfoveal and parafoveal areas. Abnormalities in the subfoveal and parafoveal reflexes were defined as (1) the presence of a hyperreflective or hyporeflective reflex in the subfoveal area (n = 17) and (2) the presence of a hyperreflective or hyporeflective reflex in the parafoveal area (n = 9). Some patients had more than one macular disorder. Figure 3 shows the systemic and ocular history distribution.

**Table 1:** The number and frequency for the three cataract morphologies stratified by LOCS III grades.

LOCS III	NO	C	P
≤2.5	79 (10.6%)	230 (25.8%)	132 (45.5%)
2.6–3.5	237 (31.7%)	218 (24.4%)	85 (29.3%)
3.6–4.5	334 (44.7%)	227 (25.4%)	56 (19.3%)
4.6–5.5	87 (11.6%)	219 (24.6%)	18 (6.2%)
>5.5	11 (1.5%)	NA	NA
Total	748	894	291

NO = Nuclear opalescence; C = Cortical; P = Posterior subcapsular; NA = Not Available.

**Figure 4:**
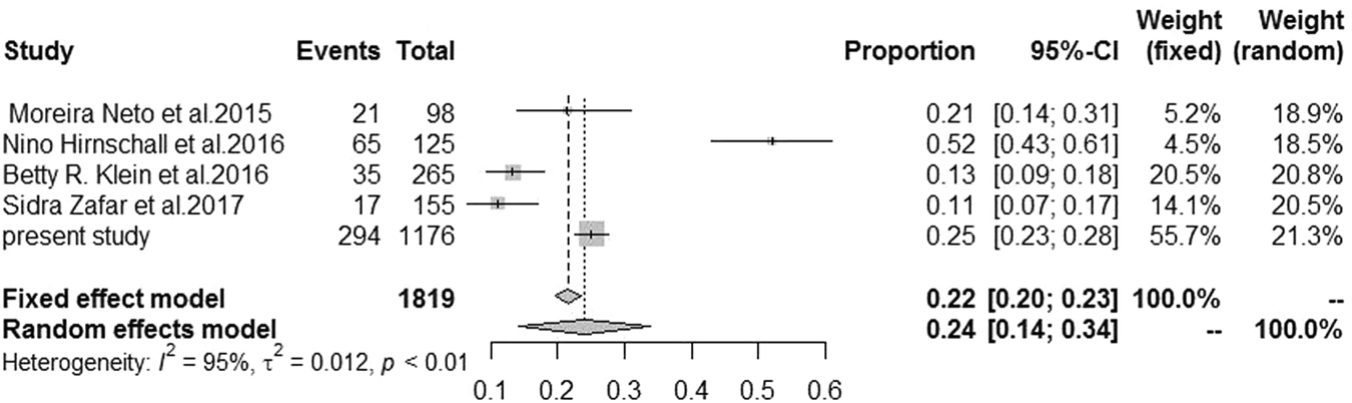
The results of Meta-Analysis.

We summarized the characteristic of the previous studies on the prevalence of macular diseases detected by OCT. Four separate studies plus the present study, encompassing a total of 1819 eyes (Table 2), were finally meta-analyzed, with one study conducted on Brazil, one on Austria, one on USA, and one on Pakistan. All the studies reported different prevalence of macular pathologies in patients before routine cataract surgery. All the studies performed the macular imaging using SD-OCT, with the exception of SS-OCT in Sidra Zafar *et al*. and binocular indirect ophthalmoscopy in Moreira Neto *et al*. The pooled prevalence rate of macular diseases detected by OCT was 0.24 (95% CI 0.14–0.34) using meta-analysis (Fig. 4). This analysis revealed significant heterogeneity across studies (P < 0.01).

**Table 2:** Primary research in the literature and the present research.

Author	Number of eyes	Abnormal number of eyes (%)	Device	Country	Publication year
Moreira Neto *et al*.	98 eyes	21 (21.4%)	SD-OCT	Brazil	2015
Nino Hirnschall *et al*.	125 eyes	65 (54.2%)	SD-OCT	Austria	2016
Betty R. Klein *et al*.	265 eyes	35 (13.2%)	SD-OCT	USA	2016
Sidra Zafar *et al*.	155 eyes	17 (10.9%)	SS-OCT	Pakistan	2017
present study	1176 eyes	294 (25.0%)	SD-OCT	China	NA

SD-OCT = Spectral-domain optical coherence tomography; SS-OCT = Swept-source optical coherence tomography.

## Discussion

Cataract remains a leading cause of visual impairment worldwide, especially in developing countries such as China, which accounts for one-fifth of the global population^8^. In developed countries, cataracts account for only 5% of cases of blindness but are responsible for 50% of cases of blindness in developing countries^9^. Cataract and retinal disease may progress concurrently in the ageing population, as in myopia, or in other cases. Cataract development may be accelerated as a result of the retinal disease process or as a sequela of corticosteroid treatment, as in diabetic retinopathy and uveitis^10^. We found that the prevalence of cortical cataract (90.1%) was higher than any nuclear cataract (75.4%), and the PSC was the least common type (29.3%). Combined cortical and nuclear cataract was the most common cataract type (45.5%) in Our study, consistent with findings reported in other studies^8–10^.

Phacoemulsification with IOL implantation has made significant advancements in the past few decades, resulting in improved refractive outcomes and greater patient satisfaction. However, not everyone can achieve perfect visual activity after cataract surgery because of different retinopathies, especially maculopathies sheltered by cataract.

To our knowledge, this is the first retrospective cross-sectional study utilizing SD-OCT screening for macular pathology in patients from a developing country before routine cataract surgery. We used SD-OCT with the TruTrack eye-tracking system and 18-line radial scanning (18 B-scans with 20-degree interscan spacing, 6-mm diameter) to ensure no omission of macular lesions^11^. Moreira Neto *et al*.^12^ investigated 98 patients undergoing cataract surgery; they diagnosed preoperative maculopathies in 21.4% of the patients with SD-OCT, which was a larger percentage than that detected with binocular indirect ophthalmoscopy (11.2%). In that study, patients with clinically detectable macular pathologies also were included. In a prospective study of 155 patients undergoing cataract surgery, swept-source optical coherence tomography (SS-OCT) diagnosed preoperative maculopathies in 10.9% of the patients^4^, similar to study findings reported by Klein *et al*.^5^. Pathological macular findings were detected using SD-OCT scans in 294 cases (25%), whereas 698 cases (59.35%) presented a normal macula in our study. These three studies excluded patients with clinically detectable macular pathologies. Our study also excluded patients with certain clinical macular pathologies (Table 2). Combined with the meta-analysis of these studies, OCT examination of patients preparing for cataract surgery can allow the surgeon and patient to find macular diseases, to understand the prognosis of vision, and to avoid some unnecessary medical suffering from disputes, so macular OCT examination before routine cataract surgery is very effective and necessary.

The three most common macular disorders observed were ERM (n = 130), followed by myopic atrophy (n = 61) and DSM with pathologic myopia (n = 32). The latter two macular diseases were definitely myopia-associated complications, and ERM is associated with age and myopia. DSM is a forward bulging of the macula within the posterior staphyloma in highly myopic eyes. Gaucher and associates first reported the diagnosis of DSM^13^. Its prevalence was reported to be 10.7% in a European study and 9.3% in a Japanese study^14^. We have not found epidemiological studies on DSM reporting on Chinese cohorts. We observed 32 eyes (2.72%) with DSM and pathologic myopia in our study, which was a lower percentage than the results obtained in other research.

ERM, also called cellophane maculopathy or macular pucker, is a disorder of the vitreomacular interface that can cause visual impairment and even metamorphopsia. SD-OCT has proven to be more sensitive and objective than other clinical examination devices for the diagnosis and classification of numerous disorders of the vitreomacular interface, including ERM^15^. In recent years, research among multiethnic groups provided population-based prevalence data on ERM. These studies revealed that the prevalence of ERM varied from 2.2% to 34.1% depending on the population studied^16–20^. It has been suggested that the prevalence of ERM is lower in Asians compared with that in whites. The Beijing Eye Study reported a relatively low prevalence of ERM, which was 2.2% in participants aged ≥40 years^20^. In the Handan Eye Study, ERMs affected 3.4% of the population aged ≥30 years living in rural China^17^. Recently, the Jiangning Eye Study reported that the prevalence of ERM in mainland Chinese individuals 50 years of age or older was estimated to be 7.3% after age and sex standardization according to the 2010 Chinese census population^19^; this value is higher than those from the two previously reported studies in mainland China, possibly because the census population was older than in the two previous studies. All three studies concluded that the prevalence of ERM was associated with age and myopia. We diagnosed 130 eyes (11.05%) with macular ERM from SD-OCT scans that had not been detected in a subjective dilated fundus examination, which accounted for almost one-half (44.22%) of all abnormalities in the diseased eyes. The patients with an ERM ranged in age from 47 to 89 years. The mean age of patients with an ERM was 70.46 ± 9.67 years and many of these diseased eyes had a history of myopia (n = 45, 34.62%). In comparison, age-related degenerative changes were the most common macular pathology detected by SD-OCT in a study of a Western population. This is possibly because ERM is more prevalent in myopic populations, and the Chinese were most likely to be affected by myopia^21^.

Abnormal scans were more common in patients with myopia (120, 40.82%), those who had previously undergone intraocular surgery (24, 8.16%) or endophotocoagulation (13, 4.42%), and those with glaucoma (17, 5.78%), retinal vasculopathy (15, 5.1%) or uveitis (4, 1.36%). The most common ocular history was myopia, including high myopia (89, 0.82%). In the past decades, the prevalence of myopia in east and southeast Asian countries, such as Singapore, China, Japan and Korea, has increased rapidly^22^. Similar trends have also been reported in Western populations^23^. Myopia has become a major public health concern, particularly because a significant proportion of individuals with high myopia develop pathological signs and blinding complications that are not preventable with optical correction^24^.

Thanks to the development of optical coherence tomography technologies, macular pathogeneses have been described in detail, which usually cannot be achieved using a traditional dilated fundus examination^7^. OCT has become fundamental in the diagnosis and management of myopia-related complications^25^. Nowadays, patients with cataract have increasingly high expectations regarding postoperative visual outcomes. Moreover, the myopic population, and especially the population with high myopia, continues to increase in China. Many studies have demonstrated that OCT is essential and advantageous for the early diagnosis, prevention, treatment and visual prognosis of macular diseases, especially myopia-associated complications, including myopic CNV, MH, DSM and so on^26–29^. By performing preoperative SD-OCT scanning, clinicians have prevented unanticipated postoperative retina consultations for pre-existing macular disease in our population with cataract.

Although SD-OCT is advantageous in resolving the different layers of the retina, and even the choroid^30^, it lacks the lateral resolution to resolve blood vessels and the cellular details of each layer^31^. More recently, the en face OCT device has also become desirable, and images slices of tissue at orientations perpendicular to the optic axis in real time. This is extremely important in studies of the eye. It combines OCT imaging with noninvasive angiography, which allows the blood vessels and blood flow to be determined without the need for formal fluorescein and indocyanine green angiography and has been used in many retinal vasculopathies. With ever-evolving techniques, we have the ability to learn more about the ultrastructure and vasculature of the retina and choroid and to detect pathological changes in the early stages.

There were specific limitations in our research. First, this was descriptive research designed to evaluate the benefit of SD-OCT as a strategy for recognizing occult macular disorders preoperatively in patients scheduled for routine cataract surgery. We did not analyse visual acuity or contrast sensitivity before or after surgery to determine which macular pathologies should be excluded from surgical treatment. The diagnoses of systemic disease histories were based on self-report, medical treatment history and clinical findings; we did not obtain detailed objective laboratory data or perform examinations.

In summary, we recommend routine preoperative macular OCT scanning in patients undergoing routine cataract surgery to evaluate visual outcomes, especially in the presence of myopia and in those considering advanced-technology IOL implantation. However, further investigation is needed to compare various OCT technologies for preoperative assessments before routine cataract surgery, and a study comparing macular structural abnormalities pre- and postoperatively is also needed.

## Methods

One-thousand-one-hundred-seventy-six consecutive subjects underwent comprehensive eye testing including corrected Snellen visual acuity, slit-lamp biomicroscopy and dilated fundus examination between January and June of 2016 in the Department of Ophthalmology, Wuxi No. 2 Hospital, Wuxi, Jiangsu Province, China. This retrospective reserch included patients with different grades of cataract and cataract classification was commonly assessed by Lens Opacities Classification System III grading scale^32^. Each patient was examined on a slit-lamp biomicroscope by an experienced ophthalmologist after pupil dilation with 0.5% tropicamide. All patients who were diagnosed with clinically visible macular abnormalities were excluded, such as macular hemorrhage, macular exudation and so on.

**Figure 5:**
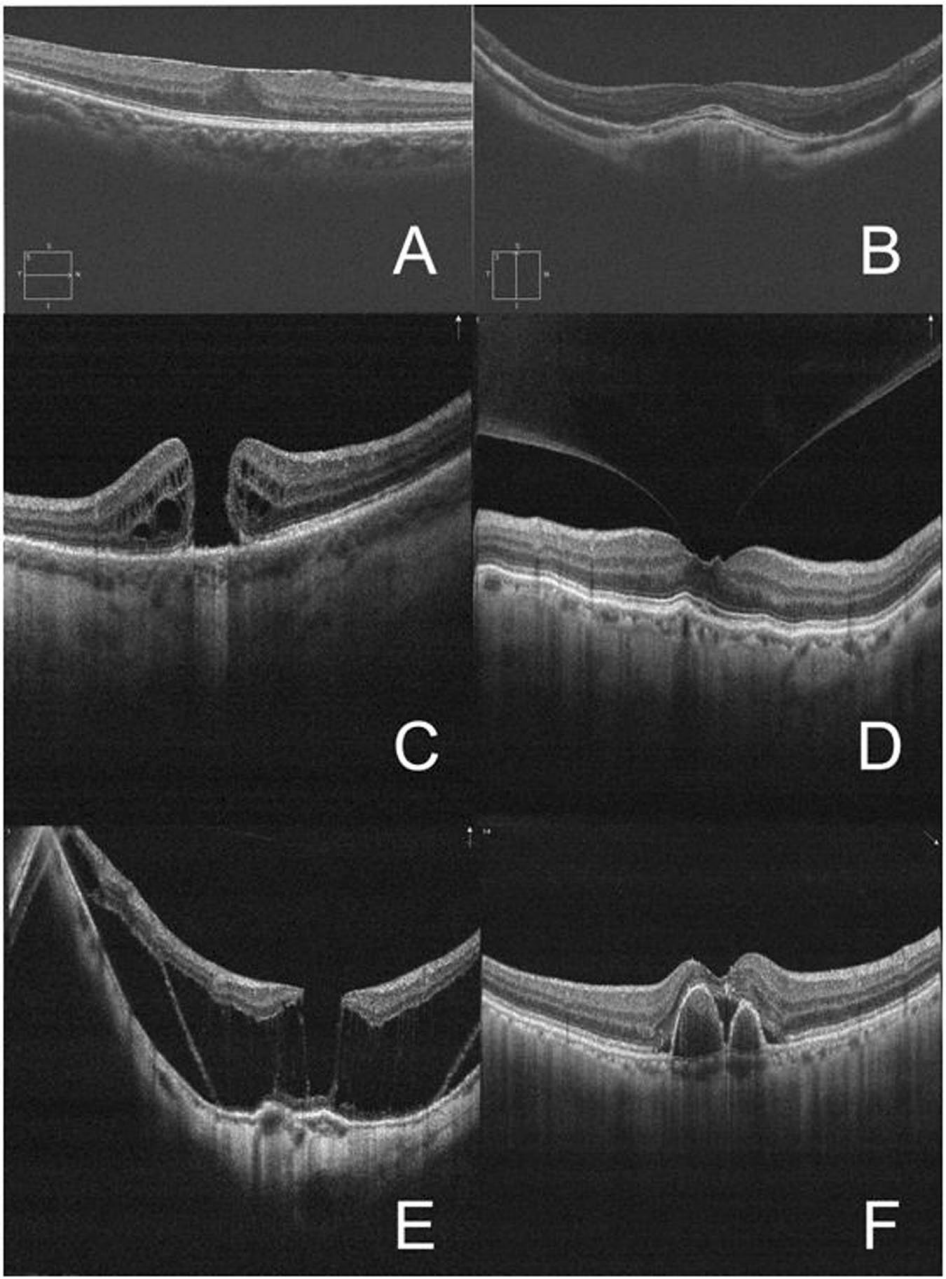
(**A**) Epiretinal membrane (N3C3); (**B**) dome-shaped macula with pathologic myopia (N3C2P1); (**C**) macular hole (N3C2); (**D**) vitreomacular traction syndrome (N3C2); (**E**) macular retinoschisis (N3P1); (**F**) polypoidal choroidal vasculopathy (N3C3).

A high-speed 840-nm-wavelength SD-OCT instrument was used to scan the macula during preoperative evaluations of all patients scheduled for cataract surgery through dilated pupils (RTVue XR Avanti; Optovue, Inc, Fremont, California, USA). All OCT scans were performed by an experienced operator (T.T.C). It requires a pupil size of 2.5 mm or larger for imaging, no luminance room conditions limitation. We used the SD-OCT radial scan protocol centred on the macula with the TruTrack eye-tracking system and 18-line radial scanning (18 B-scans with 20-degree interscan spacing, 6-mm diameter). Each participant’s head was fixed on the sustainer, upright with the eye focusing on an internal fixation target without blinking and eye movement while 18 radial scans were performed, and we selected the highest quality picture of macular disorders. All scans were reviewed by an experienced vitreoretinal specialist for macular pathology (Fig. 5). The vitreoretinal specialist was familiar with OCT scans and pre-assessment visits before cataract surgery. In this analysis, we focused on the incidence of abnormal macular SD-OCT scans, and prevalences in subgroups with abnormal scans according to age; sex; systemic history including hypertension and diabetes; ocular history including myopia, history of intraocular surgery and so on. Diagnoses of ocular and systemic diseases were based on self-reported histories, medical treatment histories and clinical findings.

The meta-analysis is reported in accordance with the Preferred Reporting Items for Systematic Reviews and Meta-analyses guideline^33^. Relevant studies were identified by systematically searching the PubMed, Embase, Web of Science, and Chinese Biomedicine databases from its inception up to September 2017. The search results were supplemented by reviews of reference lists for all relevant studies and review articles. No minimum number of patients was required for the meta-analysis.

Studies qualified for the study if they met the following criteria: retrospective cross-sectional study utilizing OCT screening for occult macular pathology in patients who are scheduled for routine cataract surgery.

The statistical analysis was performed by R software (version 3.1.1). The pooled prevalence estimates of macular diseases based on all studies were calculated assuming a random-effects model with inverse-variance weighting using DerSimonian and Laird’s method^34^. Heterogeneity across all eligible comparisons was estimated using a chi-square test. Heterogeneity was considered significant at P < 0.10. Potential publication bias was determined using the Egger regression asymmetry test. A P value less than 0.05 was considered statistically significant.

## References

[1] Zhao J (2010). Prevalence of vision impairment in older adults in rural China: the China Nine-Province Survey. Ophthalmology..

[2] Tang Y (2015). Prevalence and causes of visual impairment in a Chinese adult population: the Taizhou Eye Study. Ophthalmology..

[3] Zhu RR, Shi J, Yang M, Guan HJ (2016). Prevalences and causes of vision impairment in elderly Chinese: a socioeconomic perspective of a comparative report nested in Jiangsu Eye Study. Int. J. Ophthalmol..

[4] Zafar S, Siddiqui MAR, Shahzad R, Shahzad MH (2017). Swept-source optical coherence tomography to screen for macular pathology in eyes having routine cataract surgery. J. Cataract Refract. Surg..

[5] Klein BR, Brown EN, Casden RS (2016). Preoperative macular spectral-domain optical coherence tomography in patients considering advanced-technology intraocular lenses for cataract surgery. J. Cataract Refract. Surg..

[6] Hirnschall N, Leisser C, Radda S, Maedel S, Findl O (2016). Macular disease detection with a swept-source optical coherence tomography-based biometry device in patients scheduled for cataract surgery. J. Cataract Refract. Surg..

[7] Meuer SM (2015). The epidemiology of vitreoretinal interface abnormalities as detected by spectral-domain optical coherence tomography: the Beaver Dam Eye Study. Ophthalmology..

[8] Tang Y (2016). Prevalence of age-related cataract and cataract surgery in a Chinese adult population: the Taizhou Eye Study. Invest. Ophthalmol. Vis. Sci..

[9] Khanna R, Pujari S, Sangwan V (2011). Cataract surgery in developing countries. Curr. Opin. Ophthalmol..

[10] Ali Khan M, Skidmore K, Ho AC (2015). Perioperative retina evaluation of the cataract surgery patient. Curr. Opin. Ophthalmol..

[11] Adam MK, Rayess N, Rahimy E, Maguire JI, Hsu J (2015). Radial versus raster spectral-domain optical coherence tomography scan patterns for detection of macular fluid in neovascular age-related macular degeneration. Br. J. Ophthalmol..

[12] Moreira Neto CA, Moreira Júnior CA, Moreira AT (2015). Optical coherence tomography in patients undergoing cataract surgery. Arq. Bras. Oftalmol..

[13] Gaucher D (2008). Dome-shaped macula in eyes with myopic posterior staphyloma. Am. J. Ophthalmol..

[14] Caillaux V (2013). Morphologic characterization of dome-shaped macula in myopic eyes with serous macular detachment. Am. J. Ophthalmol..

[15] Hwang JU (2012). Assessment of macular function for idiopathic epiretinal membranes classified by spectral-domain optical coherence tomography. Invest. Ophthalmol. Vis. Sci..

[16] Aung KZ (2013). The prevalence and risk factors of epiretinal membranes: the Melbourne Collaborative Cohort Study. Retina..

[17] Duan XR (2009). Prevalence and associations of epiretinal membranes in a rural Chinese adult population: the Handan Eye Study. Invest. Ophthalmol. Vis. Sci..

[18] Ng CH (2011). Prevalence and risk factors for epiretinal membranes in a multi-ethnic United States population. Ophthalmology..

[19] Ye H (2015). Prevalence and associations of epiretinal membrane in an elderly urban Chinese population in China: the Jiangning Eye Study. Br. J. Ophthalmol..

[20] You Q, Xu L, Jonas JB (2008). Prevalence and associations of epiretinal membranes in adult Chinese: the Beijing eye study. Eye (Lond)..

[21] Pan CW (2013). Prevalence of refractive errors in a multiethnic Asian population: the Singapore epidemiology of eye disease study. Invest. Ophthalmol. Vis. Sci..

[22] Pan CW, Ramamurthy D, Saw SM (2012). Worldwide prevalence and risk factors for myopia. Ophthalmic Physiol. Opt..

[23] Lee KE, Klein BE, Klein R, Wong TY (2002). Changes in refraction over 10 years in an adult population: the Beaver Dam Eye study. Invest. Ophthalmol. Vis. Sci..

[24] Saw SM, Gazzard G, Shih-Yen EC, Chua WH (2005). Myopia and associated pathological complications. Ophthalmic Physiol. Opt..

[25] Cicinelli MV, Pierro L, Gagliardi M, Bandello F (2015). Optical coherence tomography and pathological myopia: an update of the literature. Int. Ophthalmol..

[26] Chan NS, Teo K, Cheung CM (2016). Epidemiology and diagnosis of myopic choroidal neovascularization in Asia. Eye Contact Lens..

[27] Itakura H, Kishi S, Li D, Akiyama H (2013). Observation of posterior precortical vitreous pocket using swept-source optical coherence tomography. Invest. Ophthalmol. Vis. Sci..

[28] Lin CW, Ho TC, Yang CM (2015). The development and evolution of full thickness macular hole in highly myopic eyes. Eye (Lond)..

[29] Tamura N, Sakai T, Tsuneoka H (2014). Spontaneous resolution of foveal detachment in dome-shaped macula observed by spectral domain optical coherence tomography. Clin. Ophthalmol..

[30] Zhang Z (2016). The effect of topical atropine on the choroidal thickness of healthy children. Sci. Rep..

[31] Jonnal RS (2014). The cellular origins of the outer retinal bands in optical coherence tomography images. Invest. Ophthalmol. Vis. Sci..

[32] Chylack LT (1993). The lens opacities classification system III. Arch Ophthalmol..

[33] Moher D, Liberati A, Tetzlaff J, Altman DG (2009). Preferred reporting items for systematic reviews and meta-analyses: the PRISMA statement. J Clin Epidemiol..

[34] DerSimonian R, Laird N (1986). Meta-analysis in clinical trials. Control Clin Trials.

